# R2DRV: study protocol for longitudinal assessment of driving after mild TBI in young drivers

**DOI:** 10.1186/s40621-024-00493-6

**Published:** 2024-03-13

**Authors:** Jingzhen Yang, Despina Stavrinos, Thomas Kerwin, Sylvie Mrug, Michael Tiso, Benjamin McManus, Cameron G. Wrabel, Christopher Rundus, Fangda Zhang, Drew Davis, Erin M. Swanson, Brett Bentley, Keith Owen Yeates

**Affiliations:** 1 Center for Injury Research and Policy at the Abigail Wexner Research Institute, Nationwide Children’s Hospital, 700 Children’s Drive – RBIII, Columbus, OH 43205 USA; 2 Department of Pediatrics, The Ohio State University, Columbus, OH USA; 3 Institute for Social Science Research, The University of Alabama, ISSR 107, Box 870216, Tuscaloosa, AL 35487 USA; 4 Department of Psychology, University of Alabama at Birmingham, Birmingham, AL USA; 5 The Ohio State University Driving Simulation Laboratory, Columbus, OH USA; 6 Department of Sports Medicine, The Ohio State University, Columbus, OH USA; 7 Division of Pediatric Rehabilitation Medicine, Department of Pediatrics, University of Alabama at Birmingham, Birmingham, AL USA; 8 Department of Family, Internal, and Rural Medicine, The University of Alabama, Tuscaloosa, AL USA; 9 Department of Psychology, Alberta Children’s Hospital Research Institute, and Hotchkiss Brain Institute, University of Calgary, Calgary, AB Canada

**Keywords:** Traumatic brain injury, Young driver, High-fidelity driving simulator, Return to drive, Driving performance

## Abstract

**Background:**

Mild traumatic brain injury (mTBI) and traffic-related injuries are two major public health problems disproportionately affecting young people. Young drivers, whose driving skills are still developing, are particularly vulnerable to impaired driving due to brain injuries. Despite this, there is a paucity of research on how mTBI impacts driving and when it is safe to return to drive after an mTBI. This paper describes the protocol of the study, R2DRV, Longitudinal Assessment of Driving After Mild TBI in Young Drivers, which examines the trajectory of simulated driving performance and self-reported driving behaviors from acutely post-injury to symptom resolution among young drivers with mTBI compared to matched healthy drivers. Additionally, this study investigates the associations of acute post-injury neurocognitive function and cognitive load with driving among young drivers with and without mTBI.

**Methods:**

A total of 200 young drivers (ages 16 to 24) are enrolled from two study sites, including 100 (50 per site) with a physician-confirmed isolated mTBI, along with 100 (50 per site) healthy drivers without a history of TBI matched 1:1 for age, sex, driving experience, and athlete status. The study assesses primary driving outcomes using two approaches: (1) high-fidelity driving simulators to evaluate driving performance across four experimental study conditions at multiple time points (within 96 h of injury and weekly until symptom resolution or 8 weeks post-injury); (2) daily self-report surveys on real-world driving behaviors completed by all participants.

**Discussion:**

This study will fill critical knowledge gaps by longitudinally assessing driving performance and behaviors in young drivers with mTBI, as compared to matched healthy drivers, from acutely post-injury to symptom resolution. The research strategy enables evaluating how increased cognitive load may exacerbate the effects of mTBI on driving, and how post-mTBI neurocognitive deficits may impact the driving ability of young drivers. Findings will be shared through scientific conferences, peer-reviewed journals, and media outreach to care providers and the public.

## Background

Mild traumatic brain injuries (mTBI) and traffic-related injuries are two major public health problems that disproportionately affect teens and young adults (Patricios et al. 2022; Centers for Disease Control and Prevention 2018; Institute and for Highway Safety (IIHS). 2021; Centers for Disease Control and Prevention 2018). Returning to driving after an mTBI is a common goal for many teens and young adults as they return to their daily activities (Preece et al. 2013; Jain et al. 2021), but guidance is lacking on when it is safe to do so (Christensen and McGrew 2019; Sarmiento et al. 2021). When young drivers can resume driving is of particular concern because they have the highest rate of mTBI (Patricios et al. 2022; Centers for Disease Control and Prevention 2018; Santana et al. 2020) and the highest crash rate of all age groups (Institute and for Highway Safety (IIHS) 2021; Centers for Disease Control and Prevention 2018; Mayhew et al. 2003; MacDonald et al. 2018).

Driving is a complex task requiring motor coordination, visual perception, and higher-order cognition, all of which can be impaired by a brain injury (Strayer et al. 2015; Engstrom et al. 2005). Adults with mTBI exhibit slower responses and difficulties in driving tasks compared to adults with orthopedic injury (Baker et al. 2015; Preece et al. 2010). These deficits may be more pronounced in young drivers due to their developing brains and growing driving skills (Kerwin et al. 2023; Schmidt et al. 2023). Furthermore, mTBI can reduce mental resources available for complex tasks like driving (Strayer et al. 2013; Grady et al. 2012). Because young drivers have not yet internalized the most basic driving tasks, they may be more vulnerable to interference by other cognitive demands. Thus, mTBI may have a greater effect on young drivers’ driving performance than their older adult counterparts (Taylor et al. 2013; D'Silva et al. 2021).

Current clinical practice guidelines in Canada and Australia recommend “no driving within 24 h of an mTBI,” but these guidelines are neither evidence-based nor specific to young drivers (Schmidt et al. 2017; Marshall et al. 2012; Motor Accidents Authority of New South Wales 2008). It is crucial to understand how mTBI affects young drivers both immediately after injury and during recovery, considering the significant reduction in mental resources available for driving tasks (Kerwin et al. 2023; Schmidt et al. 2023; McDonald et al. 2021; Guinosso et al. 2016; Rivara et al. 2023). This knowledge will help inform clinical decisions regarding when it is safe for young drivers to resume driving after an mTBI.

## Objectives

The objectives of the Longitudinal Assessment of Driving After Mild TBI in Young Drivers (R2DRV) study are to assess (1) the trajectory of driving performance and behaviors in young drivers with mTBI, as compared to matched healthy drivers, from acutely post-injury to symptom resolution; (2) the effect of increased cognitive load on driving performance and behaviors in young drivers with and without mTBI; and (3) the extent to which differences in driving performance and behaviors between young drivers with and without mTBI, particularly under increased cognitive load, are mediated by acute post-injury neurocognitive function. We hypothesize that (1) simulated driving performance (e.g., braking reaction time, standard deviation of speed) and self-reported driving behavior will improve over time for young drivers with mTBI from the acute post-injury phase to symptom resolution, but will remain unchanged for healthy young drivers; (2) differences in driving performance between mTBI and healthy comparison groups will be more pronounced under increased cognitive load, defined as performing a concurrent task while driving in a high-fidelity driving simulator (Strayer et al. 2015, 2013), with the group difference being largest during the acute post-injury phase and decline over the course of recovery; and (3) post-injury neurocognitive function will mediate the effect of mTBI on driving performance, with a stronger effect under increased cognitive load and during the acute post-injury phase.

## Methods

### Study design

**Fig. 1:**
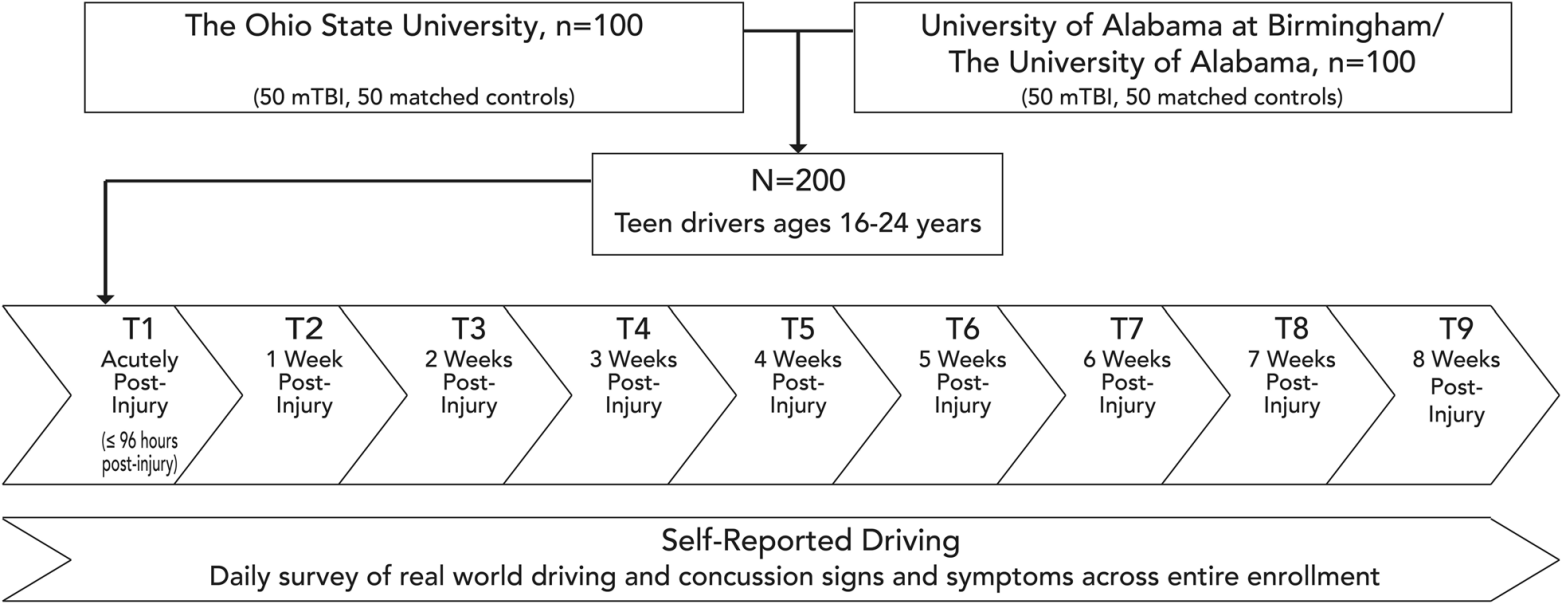
Study Design and Participant Flow

This is a multi-site, longitudinal observation study led by two co-principal investigators that aims to evaluate driving in young drivers with mTBI from the acute post-injury phase (≤ 96 h post-injury) to symptom resolution, compared to healthy drivers (no history of TBI) matched at a 1:1 ratio for age, sex, driving experience, and athlete status (Fig. 1). Matching participants on these characteristics aims to recruit groups that are comparable on key characteristics except mTBI, with the goal of reducing potential group differences on confounding variables. The study aims to enroll 200 young drivers aged 16 to 24, 100 with mTBI (50 per site) and 100 without mTBI (i.e., healthy drivers; 50 per site) from two study sites (100 drivers per site): Birmingham/Tuscaloosa, AL and Columbus, OH. Following the relocation of one of the co-principal investigators, all research activities initially based at the University of Alabama at Birmingham (UAB) were transferred to the University of Alabama (UA) at Tuscaloosa.

Recruitment takes place both pre-injury at university and high school sports team meetings and post-injury at concussion clinics and emergency departments affiliated with the universities at the two sites. After receiving physician confirmation of an isolated mTBI, injured participants complete the first assessment on a driving simulator within 96 h of injury, followed by weekly assessments until symptom resolution, defined as post-concussion symptom scores of 5 or lower for two consecutive days (Leddy et al. 2019), or a maximum of 8-weeks post-injury. The final assessment is conducted one week after symptom resolution. This assessment schedule ensures data are collected from the acute post-injury phase to one week after symptom resolution. Matched healthy drivers complete the same number of driving assessments as the matched index mTBI cases.

At each driving assessment, all participants complete four scenarios in a randomly ordered 2 × 2 design for a critical event condition (Event vs. No Event) and a cognitive load condition (Load vs. No Load). Neurocognitive testing is conducted at each in-person assessment (up to 9 time points). Daily surveys of self-reported driving behaviors and post-mTBI symptoms (both mTBI and healthy drivers) are collected throughout each participant’s enrollment period using Research Electronic Data Capture (REDCap), a secure web-based application. This study has received ethical approval from the Institutional Review Board (IRB) at the leading institution, Nationwide Children’s Hospital (NCH), as a single IRB for Multi-Site Research (Institutional Review Board at NCH, IRB16-01226). Participation in the study does not pose significant risks and does not affect participant care.

## Participants

The study will enroll a total of 200 young drivers aged 16 to 24 years (100 mTBI and 100 matched healthy drivers). This age range was selected because the minimum age to drive unsupervised is 16 in Ohio and Alabama, the two study sites, and the brain is not fully developed until the mid-20s. An mTBI is characterized according to the World Health Organization’s definition (Carroll et al. 2004) as a brain injury resulting from mechanical energy to the head from external physical forces with one or more of the following acute conditions confirmed by a physician: (i) confusion or disorientation, (ii) loss of consciousness ≤ 30 min, (iii) post-traumatic amnesia < 24 h, (iv) Glasgow Coma Scale score of 13–15 after 30 min or more post-injury, and/or (v) other transient neurological abnormalities such as focal signs or intracranial lesion not requiring surgery (Carroll et al. 2004). Eligible participants must have a valid driver’s license. Exclusion criteria include if the injury (1) requires surgery or hospitalization, (2) is related to a motor vehicle collision, (3) is intentional (i.e., assault, abuse, or self-harm), (4) is associated with illicit drug or alcohol use, (5) involves a penetrating injury, or (6) leads to any comorbidities affecting the individual’s ability to drive (e.g., post-traumatic stress disorder, or conditions that affect eyesight, dominant arm, or right leg). Two physician co-investigators, one at each study site, review each case to confirm eligibility.

## Study procedures

### Recruitment

Information about the study is posted in school newsletters and on school websites through our collaborations with large school systems at each site, as well as in emergency medicine departments and concussion clinics. Eligible young drivers are recruited pre-injury from sports teams and school events, and post-injury from the emergency medicine departments and concussion clinics at the hospitals affiliated with The Ohio State University (OSU) or UAB/UA.

For pre-injury recruitment, the research staff presents the study information at scheduled pre-season meetings. Consent/assent to contact documents are collected along with participant sex, age, athlete status, and type/month of licensure to create a sampling pool for potential cases and matched healthy drivers. For post-injury recruitment, both sites’ athletic departments provide referrals using an injury documentation app (e.g., Healthy Roster).

### mTBI cases

Upon receiving a referral of a confirmed diagnosis of an isolated mTBI from a physician/physician office or an athletic trainer, the research staff contacts the young driver (or parent if under 18 years old) to confirm interest, review eligibility and study protocol, answer questions, and schedule an in-person meeting within 96 h of injury for the first driving simulator assessment. The researcher obtains consent/assent either at the first contact or before the scheduled first assessment begins.

#### Matched healthy drivers

Healthy (not injured, no history of TBI) participants are selected from the sampling pool, matched to an index case, and contacted after case identification to consent and complete all weekly assessments. Matching variables include sex, age (± 6 months), athlete status (yes/no; “athlete” defined as engaged in organized sport at time of enrollment), type of licensure, and months since receiving licensure (± 3 months). If they decline participation, the next closest matched driver is selected until a driver is enrolled. If a matched healthy driver becomes injured during follow-up, they become an mTBI case and a new matched healthy driver is recruited. Data collected prior to injury are included in the analysis as a healthy driver.

### Retention plan

To maximize participant retention, we employ several strategies: 1) maintaining a diverse research staff; 2) collecting participants’ personal information and contact information; 3) closely monitoring daily surveys and reaching out to participants if they fail to complete them; 4) sending automated daily text messages with survey links; 5) offering compensation for transportation for an mTBI participant’s first visit to the simulator facilities; and 6) providing compensation to enhance engagement in the study.

## Study data collection

Participants complete up to 9 assessments in a high-fidelity driving simulator. The first assessment occurs within 96 h of injury for mTBI participants or within one week of identification for healthy drivers. Weekly assessments continue until one week after symptom resolution or the maximum of 8 weeks post-injury is met. Each assessment consists of surveys before driving, neuropsychological testing [i.e., ImPACT Quick Test and National Institutes of Health (NIH) Toolbox] after driving (Schatz and Ferris 2013; Holdnack et al. 2017), and balance and vision tests conducted only during the first and final assessments. Participants are advised to report any motion sickness, dizziness, or symptom elevation. They can stop the assessment at any time. Before completing the four driving scenarios described below, participants complete an introductory practice drive (see Table 1 for key driving variables).

**Table 1:** Measures Organized by Domain, Method of Administration, and Time Point

Domain	Measures	Method of administration		Time point	
		Computer	Self-report	Baseline only	All time points
*Driving Outcomes*					
Simulated driving performance	Standard deviation of speed	X			X
	Standard deviation of lane position	X			X
	Braking reaction time	X			X
	Total braking reaction time	X			X
Real-world driving behaviors	Driving Habits Questionnaire		X		X
*Demographics*	Age, sex, race, years of driving, type/date of licensure, injury information, athletic status, history of TBI		X	X	
*mTBI-Related Variables*					
Post-mTBI symptoms	Post-Concussion Symptom Scale (PCSS)*		X		X
Neuropsychological functioning	The ImPACT Quick Test	X			X
	NIH Toolbox	X			X
Balance	Modified Balance Error Scoring System (mBESS)*		X		X
Functional Impairment	Functional Disability Inventory*		X		X
	PedsQL™*		X		X
*Secondary Variables*					
Task execution effort	NASA TLX survey		X		X
Sensation seeking	Brief Sensation Seeking Scale (BSSS)		X		X
Psychological variables	Adelaide Driving Self-Efficacy Scale (ADSES)		X		X
	Emotion Regulation Index for Children and Adolescents (ERICA)		X		X
	Barratt Impulsiveness Scale (BIS)		X		X
	Decision Making Scale		X		X
	National Longitudinal Study of Adolescent to Adult Health, Problem solving items		X		X

^*^Adopted from the Common Data Elements for Pediatric Traumatic Brain Injury

**Fig. 2:**
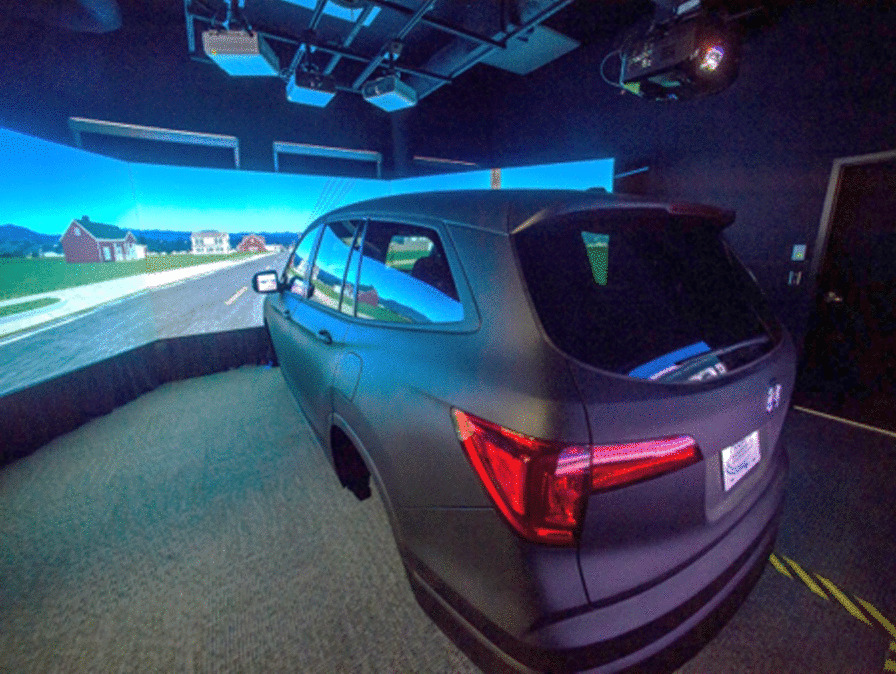
A High-fidelity Driving Simulator

*Driving Simulator.* Driving performance testing at both sites is performed on a Realtime Technologies driving simulator platform (Fig. 2). The state-of-the-art driving simulator used in this study features a full vehicle cab mounted on a motion base, providing the driver with motion cues to reduce simulator sickness and to increase realism. The simulator also includes an instrumented steering wheel, throttle, brake, gear selector, turn signals, and dashboard. The visual system consists of a front-projection screen and a rear screen, allowing the driver to see the simulated environment behind the vehicle through the center rear mirror, as well as ambient traffic through side mirror LCD screens. Full Doppler sound effects add realism to proper pass-by sounds for ambient traffic. The custom-developed driving scenarios, as described in the next section, were meticulously crafted to replicate various aspects of driving, such as traffic patterns, road conditions, and interactions with other vehicles and pedestrians, providing realistic driving experiences to participants in a safe and controlled environment.

### Driving scenarios

The driving simulator scenarios take place in daytime conditions with clear weather and traffic in the opposing direction. Environmental scenery includes trees, buildings, and road infrastructure such as intersections and highway off-ramps. Participants acclimate to the simulator environment with a 5-min practice drive and cognitive load task demonstrations.

*Experimental Simulated Drives.* Participants drive in four simulated scenarios, each lasting about 8 min. Participants begin behind a lead vehicle and are instructed to maintain a speed of 45 miles per hour without overtaking. The lead vehicle brakes randomly, requiring participants to brake to avoid a collision. The lead vehicle does not resume driving until the participant has braked. After multiple braking events, the lead vehicle pulls off to the side of the road, allowing the participant to continue down the roadway. At the end of each scenario, a recorded message instructs participants to stop, put the vehicle in park, and wait for further instructions. Transitional breaks between scenarios minimize motion sickness and fatigue. Eye tracking technology is used to measure participants’ eye movements in real-time (Taylor et al. 2013). After each scenario, participants rate their level of task execution efforts (Hart and Staveland 1988). To protect against symptom exacerbation while in the simulator, we advise participants to stop if needed.

The four scenarios involve a 2 × 2 design (1) No Cognitive Load, No Safety Critical Event; (2) No Cognitive Load, Safety Critical Event; (3) Cognitive Load, No Safety Critical Event; and (4) Cognitive Load, Safety Critical Event] and are randomly ordered for each weekly assessment such that scenarios are presented in the same order for mTBI and matched healthy drivers. The safety critical event and cognitive load conditions are described below.

### Two driving conditions with and without safety critical events

- *Condition 1—No safety critical events:* Participants perform general driving maneuvers (e.g., regulating vehicle speed and direction, lane positioning, maintaining safe following distance). There are no events requiring the participant to make any braking or evasive maneuvers.
- *Condition 2—Driving requiring immediate reaction to safety critical events*: Participants encounter three unexpected, sudden, on-screen, safety critical events requiring evasive maneuvers (e.g., braking) to avoid colliding with an object in the driving environment. For example, a car suddenly pulls onto the road at a speed of 15 miles per hour or a trash can suddenly falls onto the roadway. The three safety critical events are randomly presented in each scenario to minimize possible practice effects and anticipation.

### Two driving conditions with and without cognitive load

- *Condition 1—No cognitive load (no concurrent task):* Participants drive with no secondary cognitive tasks.
- *Condition 2—Cognitive load (while concurrently performing secondary tasks):* Participants drive while concurrently performing 2 tasks that increase cognitive load: 1) *Box Recognition–* Participants encounter half-meter-sized boxes randomly appearing above the roadway with a vertical or horizontal stripe. Participants are instructed to press a button on the steering wheel only when they see a box with a horizontal stripe; and 2) *Operation Span (OSPAN) test*—an auditory task presenting alternating math problems and short-term memory recall problems (Strayer et al. 2013, 2016). The OSPAN was recorded so both sites utilize the same OSPAN trials. The OSPAN recording asks participants to recall letters in serial order interspersed with true–false math problems (e.g., “[6/2] – 1 = 2?”, “F”, “[3 × 2] + 2 = 4?”, “J” – “Please recall the remembered letters in the correct order”). The number of trials increases before serial letter recall is queried over the duration of the scenario. Participants respond aloud with their answers (True or False for math, letter order for serial recall), which are recorded both by the tester and the simulator’s observational recording.

## Test fidelity

Testing and training protocols are shared between sites to ensure the simulation testing is delivered consistently between the two study sites as well as across and within individual participants. These strategies include detailed procedures for video recording and reviewing the driving tests, and regular bi-weekly meetings to discuss any issues encountered. Additionally, we conduct process evaluations and quality control checks for 20% of randomly selected assessments. This includes driving simulator testing, surveys, and computerized testing.

## Study variables and measures

All measures described in Table 1 have been used by the research team in previous studies and have demonstrated adequate reliability and validity with individuals with mTBI and healthy young drivers (detailed below) (Kerwin et al. 2023; Leddy et al. 2019; Yang et al. 2021). Most variables are adopted from core measures in the Common Data Elements for Pediatric TBI Studies (NINDS Common Data Elements 2023).

### Driving outcomes

*Simulated driving performance* metrics are computed across the 4 study conditions**.** Two key variables recorded continuously are: (1) standard deviation of speed, with greater fluctuation indicating inefficient driving (Evans 1991), and (2) standard deviation of lane position, a measure sensitive to the demand of secondary tasks (Evans 1991). Two key outcomes measuring driver response to safety critical events include: (1) braking reaction time, which is the time between the presentation of stimulus and the first force applied to brake (sum of neurological time + foot removal time + motion time) (Shechtman et al. 2007) and (2) total braking reaction time, which is the time between the presentation of stimulus and 200 Newtons of force applied to the brake pedal (sum of braking reaction time + time to apply 200 N of force to brake pedal) (Shechtman et al. 2007). These variables were selected based on published literature, pilot study results, and their potential association with mTBI-related neurocognitive deficits (Evans 1991; Shechtman et al. 2007).

*Self-reported driving behaviors* are measured at weekly assessments using the driving space and crashes/citations domains of the Driving Habits Questionnaire (Owsley et al. 1999**).** Participants report their driving history and driving avoidance in 9 scenarios (e.g., at night, bad weather, high traffic roads). This measure has good internal consistency (Cronbach’s α of 0.96 for driving space and 0.69 for crashes/citations) (Song et al. 2015).

*Demographics.* Participant demographic information includes age, sex, race/ethnicity, athlete status, sport(s) currently playing, time since licensure, and type/date of licensure. Injury information includes date of injury, diagnosis, previous injury (yes/no), acute signs and symptoms, mental status, and post-injury daily physical and cognitive activity including sleep. Self-reported pre- and post-injury driving history includes average hours driven per day and week and history of crash-related events (e.g., citation(s), crash(es), near crash(es)).

#### mTBI-related variables

*Post-mTBI symptoms* are assessed daily from injury to symptom resolution using the Post-Concussion Symptom Scale (PCSS) (McLeod and Leach 2012). The PCSS consists of 22 concussion symptoms/signs rated from 0 (no symptoms) to 6 (severe symptoms). The total PCSS score, ranging from 0 to 132, measures current symptom severity. The PCSS is the most common mTBI symptom rating scale, with established reliability including internal consistency (*α* = *0.93*), construct validity, and normative data (McLeod and Leach 2012).

*Neuropsychological functions* are measured at weekly assessments using the ImPACT Quick Test and NIH Toolbox (Schatz and Ferris 2013; Holdnack et al. 2017). The ImPACT Quick Test is a brief computerized cognitive test to aid in the assessment of concussions in individuals aged 12–70 years. Administered on an iPad, the ImPACT Quick Test takes 5–7 min to complete and measures attention, motor speed, and memory via three standardized percentile rank scores (Schatz and Ferris 2013). The NIH Toolbox is a multidimensional set of brief measures assessing cognitive, emotional, motor, and sensory function from ages 3 to 85 (Holdnack et al. 2017). For this study, we selected the following tests to measure processing speed, attention, visual memory, and working memory: Flanker Inhibitory Control & Attention Test, Pattern Comparison Processing Speed Test, Dimensional Change Card Sort Test, and List Sorting Working Memory Test.

*Balance* is measured at the first and final assessment using the Modified Balance Error Scoring System (mBESS) (Guskiewicz 2001). mBESS is a rapid, standardized, objective test that has been widely used following mTBI. Three different stances are held, all with hands on hips and eyes closed, for 20 s on a hard surface. Error points are given for specific behaviors including stepping, stumbling, or falling. The mBESS has shown satisfactory reliability (*ICC* = *0.7*) in youth and adolescents, with demonstrated content validity in concussed athletes (Guskiewicz 2001). Additionally, complex tandem gait is included as part of balance assessment.

*Functional impairment,* defined as the limitations or difficulties an individual experiences in performing daily activities or tasks, is evaluated at weekly assessments using the Functional Disability Inventory (FDI) (Claar and Walker 2006) and the Pediatric Quality of Life Inventory (PedsQL) (Varni and Limbers 2009). The FDI assesses participants’ perceived activity limitations attributable to mTBI and has shown high reliability (α = 0.86 - 0.91*)* and validity for adolescents (Claar and Walker 2006). The PedsQL assesses broader aspects of a participant’s quality of life, in terms of physical, emotional, social, and school functioning. The four subscales generate total, psychosocial, and physical health summary scores (Varni and Limbers 2009).

### Secondary variables

*Task execution effort, the perceived capacity to* effectively complete the task at hand, is measured following each of the 4 study drives with the NASA Task Load Index (TLX) survey (a 21-point Likert scale ranging from “very low” to “very high”) (Hart and Staveland 1988). Drivers rate their task execution effort in response to 6 questions, including ‘How mentally demanding was the task?,’ and ‘How physically demanding was the task?’ The NASA TLX has high convergent and concurrent validity, as well as test–retest reliability (r = 0.77) (Hart and Staveland 1988).

*Sensation seeking*, a trait characterized by the tendency to seek out novel, intense, and thrilling experiences, is measured at the first and final assessment using the Brief Sensation Seeking Scale (BSSS), (Zuckerman et al. 1978) an 8-item measure with adequate internal consistency (0.76) and validity in teens and young adults. Sensation seeking is consistently linked to risky driving and is more prevalent in athletes with a history of concussions (Zuckerman et al. 1978). It is treated as a potential covariate to account for risky driving behaviors in the context of return to drive.

*Psychological Variables* are measured weekly: (1) *Driving self-efficacy* (belief in ability to successfully perform driving-related tasks): Adelaide Driving Self-Efficacy Scale (ADSES, Cronbach’s α = 0.98) (George et al. 2007); (2) *Emotion regulation over the past 7 days* (ability to effectively manage and regulate emotions): The Emotion Regulation Index for Children and Adolescents (ERICA, Cronbach’s α 0.75, test–retest reliability = 0.76) (Aboulafia-Brakha et al. 2016); (3) *Impulsivity* (tendency to act without thinking): Barratt Impulsiveness Scale (BIS, Cronbach’s α = 0.77, test–retest reliability = 0.83) (Lilian and Casto 2013); (4) *Decision making* (ability to make effective and rational decisions): 8-item self-reported Decision-Making Scale (Cronbach’s α = 0.78) (Knight et al. 2014); and 5) *Problem solving* (ability to identify and define problems and generate and evaluate potential solutions): Problem-solving items from the National Longitudinal Study of Adolescent to Adult Health (National Mentoring Resource Center 2024). Some of these variables may be combined into composites based on the results of correlational and exploratory factor analysis.

## Analytic plan

### Missing data

For participants who are unable to complete all four driving experiment conditions during an assessment, the recorded driving data prior to their stop will be included in the analysis. The amount and mechanisms of missing data will be assessed. In all primary analyses, missing data will be handled with Full Information Maximum Likelihood, which estimates parameters from all available data, preserves the overall sample size, and minimizes bias (Raykov 2005). If missingness is related to the outcome variables, we will conduct sensitivity analyses using multiple imputation with a simulation-based approach from a model that describes the missing mechanism (Rubin and Schenker 1991).

*Descriptive Analysis.* We will conduct a thorough examination of all study variables (except mTBI characteristics) and compare the mTBI and matched healthy groups using t-tests (continuous variables) and chi-square tests (categorical variables) as appropriate. Distributions of continuous variables will be tested for normality, with robust estimation methods used in the main analyses if normality is violated. Bivariate associations among variables will be tested with correlations; highly correlated (|r|> 0.80) measures of similar constructs will be combined into composite variables. We will also address biological variables such as sex and age to determine whether they moderate simulated driving performance and self-reported driving behaviors. Finally, we will screen potential covariates for their distributions and relationships to independent and dependent variables of interest to guide final selection of covariates and confounders to be adjusted for in the main models. These include relevant demographic and injury characteristics, and other potential covariates, such as race/ethnicity of participant, previous injury, substance use, psychological variables, history of crashes/near crashes, and average hours driven per week.

#### Primary analyses

To characterize the trajectory of driving performance and behaviors from acutely post-injury to symptom resolution among young drivers with mTBI in comparison to healthy participants, we will define the main outcome as simulated driving performance under the ‘no cognitive load’ condition, measured by 4 key driving performance variables: standard deviation of speed, standard deviation of lane position, braking reaction time, and total braking reaction time (Table 1). We will also define a secondary outcome as self-reported driving behavior, measured as average miles driven per day since the last assessment.

We will first visually inspect the shape of individual trajectories for each driving outcome using graphing techniques (Singer and Willet 2003). We will then use a multilevel linear model for normally distributed outcomes to estimate unconditional growth models, with assessment times (i.e., up to 9 time points per person at Level 1) nested within individuals (Level 2). Time will be measured as days since the first assessment (T1), and linear and quadratic growth parameters will be tested as fixed and random effects. This method will help determine the best fitting, most parsimonious growth model for each outcome. While we hypothesize that the linear growth model will have the best fit, any significant quadratic terms will be retained and predicted by the same variables as the linear slope in the next conditional analysis. These models accommodate different numbers and spacing of assessments across participants and estimate individual intercepts and slopes of trajectories through random effects that vary across individuals.

To model the driving performance variables, an additional variable indicating the presence vs. absence of safety critical events will be added as a fixed effect at Level 1, both as a main effect and an interaction with the slope (i.e., testing if the gap between driving performance with vs. without the safety critical events changes over time). Time-varying covariates, including psychological variables from the weekly assessments, will be modeled at Level 1. mTBI status (yes vs. no) and time-invariant covariates (e.g., study site, mTBI history, sex, race/ethnicity, and driving history) will be added as fixed effects at Level 2 to predict the intercept (driving performance at T1) and slope (linear change in driving performance over time). Nonsignificant covariate effects will be omitted to enhance model parsimony. Model results will be used to estimate the timing of recovery of driving after mTBI (i.e., estimated time when the difference between mTBI cases vs. controls is zero), both overall and for subgroups defined by significant covariates (e.g., males with high T1 symptoms).

To examine the effect of cognitive load on driving performance from acutely post-injury to symptom resolution in young drivers with mTBI compared to healthy participants, we will analyze the 4 simulator driving outcomes from each study condition at each time point (Singer and Willet 2003). Using the multilevel models from Aim 1, cognitive load and cognitive load by safety critical event interaction will be added at Level 1 as fixed effects to indicate differences in driving between absence vs. presence of cognitive load when a safety critical event is absent vs. present. We will add their interactions with time (slope) to estimate changes in these effects over time. mTBI and Level 2 covariates listed in Aim 1 will be added as predictors of these 4 Level 1 effects: cognitive load, cognitive load by time, cognitive load by safety critical events, and cognitive load by safety critical events by time. As in Aim 1, non-significant covariate effects will be trimmed from the model to increase parsimony.

**Fig. 3:**
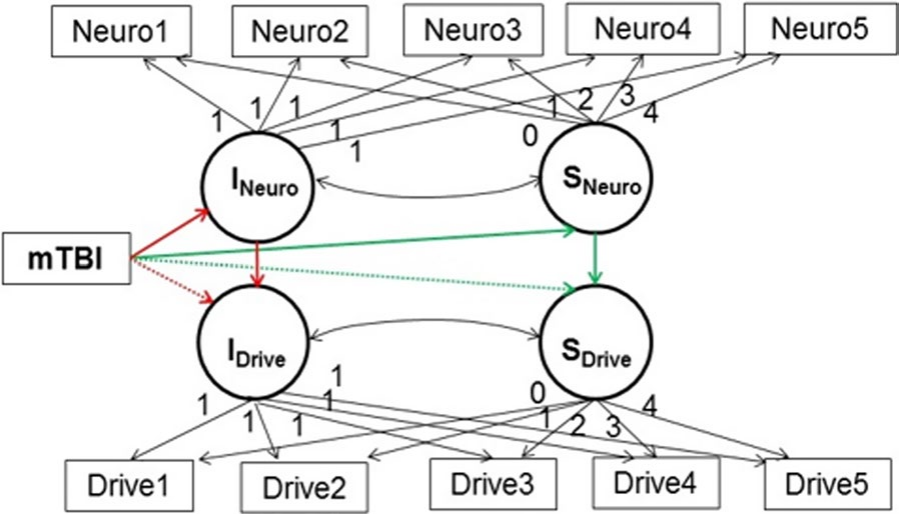
Structural Equation Model Testing Whether Neurocognitive Impairment Mediated the Effects of mTBI on Driving

To examine whether differences in driving performance between young drivers with and without mTBI, especially under increased cognitive load, are mediated by acute post-injury neurocognitive function, we will use a Structural Equation Model (Fig. 3) with driving outcomes from the most demanding driving condition (cognitive load with safety critical events) as the key outcomes. To ensure sufficient data coverage, we will only use data from the first 5 assessments for this aim.

For neurocognitive functioning, principal components analyses will be conducted first to check whether the obtained measures of motor and processing speed, attention, and memory could be summarized in fewer component scores. Based on the results, component scores will be created at each time point as an average of standardized variables that had high loadings (< 0.40) on each principal component. If the principal components analysis does not support the presence of summary dimensions, individual neurocognitive measures will be used.

To ensure sufficient degrees of freedom and facilitate model convergence, only one neurocognitive variable (or composite) and one driving outcome variable will be used in each structural equation model. The model will include two latent growth curves, one for neurocognitive functioning (processing speed, attention, memory, or their composite score) and one for driving outcomes (speed variability, lane position variability, breaking reaction time, or total breaking reaction time) across the 5 time points, characterized by two latent growth factors, intercept (T1 level) and slope (linear change over time), for each. mTBI will be included as a predictor of both intercepts and slopes, and each growth parameter for neurocognitive function will predict the same growth parameter for driving (i.e., neurocognitive intercept will predict driving intercept, neurocognitive slope will predict driving slope). Mediation will be tested with bias-corrected bootstrapping (Preacher and Hayes 2008) using 5,000 bootstrap samples for two indirect effects: 1) mTBI neurocognitive intercept driving intercept (red paths, Fig. 3; testing whether acute neurocognitive impairment mediates the effect of mTBI on acute post-injury driving performance), and 2) mTBI neurocognitive slope driving slope (green paths, Fig. 3; testing whether changes in neurocognitive function over time mediate the effects of mTBI on changes in driving over time). A similar model will be estimated using self-reported driving. A sensitivity analysis will be conducted by repeating these models with potential confounders added as time-invariant or time-varying covariates. Candidates for these covariates will include sociodemographic and injury characteristics, substance use, psychological variables, history of crashes/near crashes, and average hours driven per week, if they are related to both the neurocognitive mediator and driving outcome in preliminary analyses.

### Secondary analyses

We will conduct the following secondary analyses:

a Examine task execution effort. Self-reported task execution effort will be used to (1) validate the participant’s sensitivity to the given cognitive load by regressing self-reported task execution effort against cognitive load with adjustment for safety critical events in the multilevel models; and (2) further explore the moderating effect of cognitive load using the 2-level model described in Aim 1, replacing cognitive load (yes/no) with self-reported task execution effort.
b Examine associations of demographics and secondary variables with simulated driving performance and daily driving behaviors. Independent samples t-tests or ANOVAs (categorical demographics) and correlations (continuous variables) will examine bivariate relationships of demographics and secondary variables with driving performance and behaviors at baseline. Multiple regressions will test the effects of demographics and secondary variables on driving performance and behaviors. Bonferroni correction will be used to retain overall Type I error level of 0.05

#### Additional sensitivity analyses

We will estimate the degree of clustering within the case–control matched pairs on the four driving outcomes using Intraclass Correlation Coefficients (ICC). If any of the ICCs exceed 0.05, the analyses for that outcome will be repeated with adjustments for clustering by either adding an additional Level 3 for the matched pairs in the multilevel models (i.e., time points nested within individuals nested within matched pairs) or by adjusting standard errors using the sandwich estimator in the Structural Equation Models.

## Sample size and power

Our preliminary studies demonstrated medium to large effect sizes. Statistical power and sample size computations for the proposed analyses were calculated with an alpha equal to 0.05, statistical power ≥ 0.80, sample size of 200 (100 mTBIs and 100 controls), and attrition rate of 10%, using R packages. Multilevel models of driving outcomes (with median 5 time points; Aims 1 and 2) with up to 10 time-invariant covariates (study site, mTBI history, sex, race, ethnicity, and driving history) and 4 time-varying covariates (driving self-efficacy, emotion regulation, impulsivity, and problem solving/decision making) explaining 10–15% of outcome variance will have power of 0.92 to 0.99 to detect medium (d = 0.50) to large (d = 0.80) effects of predictors on the linear slope and power of 0.85 to 0.99 to detect medium (d = 0.50) to large (d = 0.80) effects on the quadratic slope, assuming intraclass correlation coefficient of 0.50. The power to detect medium indirect effects (beta = 0.30) with bias-corrected bootstrapping (Aim 3) is 0.85, after accounting for attrition and covariates. Based on Monte Carlo simulations, the power to detect medium-sized (d = 0.50) age and sex differences in the main models is above 0.76. Thus, the proposed sample size provides sufficient power to detect the expected medium to large effects.

## Dissemination

Our dissemination plan includes multiple strategies to reach a wide audience: (i) traditional academic outreach (e.g., publications in high-quality peer-reviewed journals and presentations at regional, national, and international conferences), (ii) media outreach and related materials (e.g., reports, newspapers, radio, TV, and social media), (iii) personal contacts (e.g., professional networks, experts in the field, practitioners), (iv) key stakeholders and organizations (e.g., sports leagues and organizations, university athletic departments, driving schools, public school systems), and (v) actionable recommendations for concussion prevention and mitigation programs. In addition, we will include a plan to communicate the aggregate study findings with study participants. These recommendations will be designed to empower patients, clinical community, and sport organizations to take proactive steps in addressing the issue.

## Discussion

This study is the first to longitudinally evaluate the impact of mTBI on the simulated driving performance and self-reported driving behaviors among young drivers from acutely post-injury to symptom resolution. It will provide critical evidence on *when* young drivers can safely resume driving after sustaining an mTBI.

The COVID-19 pandemic has posed challenges to study recruitment, such as the suspension of sporting events and closures of sport clubs due to lockdown orders. Additionally, changes in school and university class settings and sports practices have been implemented to reduce contact. To overcome these challenges, our team developed virtual tools for participant consent and enrollment. We also adjusted our eligibility criteria and extended the timeline to increase enrollment.

The study findings will have a major impact on various aspects of healthcare, policies, and practices. Specifically, this study will contribute to scientific knowledge and inform the development of clinical practice guidelines by providing critical evidence on when young drivers can safely return to drive after mTBI, how increased cognitive load may exacerbate the effects of mTBI on driving performance of young drivers, and how post-mTBI neurocognitive deficits may impact the driving ability of young drivers. For example, the study may reveal that young drivers can resume driving at 1-week post-injury with low cognitive demand, but it may take up to 3 weeks under higher cognitive demands. Moreover, the study results may be used to develop an online tool for clinicians, aiding them in determining when a patient should resume driving based on key patient characteristics (Preacher and Hayes 2008). This tool could enhance clinical decision-making and optimize patient outcomes. Additionally, the findings may inform the development of future interventions and studies that evaluate the implementation and effectiveness of clinical practice guidelines for safe driving practices among young drivers with mTBI. Ultimately, this research has the potential to contribute to a reduction in motor vehicle collisions and related injuries among young drivers following mTBI.

## Data Availability

The datasets used and/or analyzed during the current study will be available from the corresponding author on reasonable request.

## Abbreviations

mTBI: Mild traumatic brain injury
IRB: Institutional Review Board
NCH: Nationwide Children’s Hospital
NIH: National Institutes of Health
OSU: The Ohio State University
REDCap: Research Electronic Data Capture
UA: The University of Alabama at Tuscaloosa
UAB: The University of Alabama at Birmingham
ADSES: Adelaide Driving Self-Efficacy Scale
BSSS: Brief Sensation Seeking Scale
BIS: Barratt Impulsiveness Scale
ERICA: Emotion Regulation Index for Children and Adolescents
FDI: Functional Disability Inventory
mBESS: Modified Balance Error Scoring System
OSPAN: Operation Span test
PCSS: Post-Concussion Symptom Scale
PedsQL: Pediatric Quality of Life Inventory
TLX: Task Load Index
